# A pragmatic window of opportunity to minimise the risk of MRONJ development in individuals with osteoporosis on Denosumab therapy: a hypothesis

**DOI:** 10.1186/s13005-021-00280-4

**Published:** 2021-07-09

**Authors:** Giuseppina Campisi, Rodolfo Mauceri, Francesco Bertoldo, Vittorio Fusco, Alberto Bedogni

**Affiliations:** 1 Department of Surgical, Oncological, and Oral Sciences, University of Palermo, Palermo, Italy; 2 Department of Biomedical and Dental Sciences, Morphological and Functional Images, University of Messina, Messina, Italy; 3 Department of Medicine, University of Verona, Verona, Italy; 4 Oncology Unit, Azienda Ospedaliera di Alessandria SS, Antonio e Biagio e Cesare Arrigo, Alessandria, Italy; 5 Regional Center for Prevention, Diagnosis and Treatment of Medication and Radiation-Related Bone Diseases of the Head and Neck, University of Padua, Padua, Italy

**Keywords:** Medication-related osteonecrosis of the jaw, MRONJ, Denosumab, osteoporosis, Oral surgery, tooth extraction

## Abstract

**Abstract:**

Denosumab is associated with the development of medication-related osteonecrosis of the jaw (MRONJ), an uncommon but severe oral side effect with a higher prevalence in metastatic cancer patients than in patients with metabolic bone fragility. Although several oral triggers can initiate MRONJ, invasive oral treatments and tooth extraction still remain the most common precipitating event. In general, tooth extraction and oral surgery should be avoided in patients at increased risk of MRONJ, while extraction of non-restorable teeth should be performed based on specific risk reduction protocols to eliminate dental/periodontal infections, still protecting from MRONJ onset.

Based on the different pharmacological activity of denosumab and nitrogen-containing bisphosphonates, it is likely that the MRONJ risk profile of patients with osteoporosis could somewhat vary.

We hypothesize the chance to maximize the pharmacokinetic of denosumab 60 mg (Prolia®) and identify a time interval in which invasive oral treatments can ideally take place without restrictions in patients with metabolic bone fragility,

We propose that dental surgery (e.g. tooth extraction) may be safely performed without additional intra or peri-operative procedures in osteoporosis patients using denosumab provided that careful case selection, adequate communication among specialists, planning of a delayed dosing window (1-month deferral) and rigorous postoperative follow-up are granted.

**Graphical abstract:**

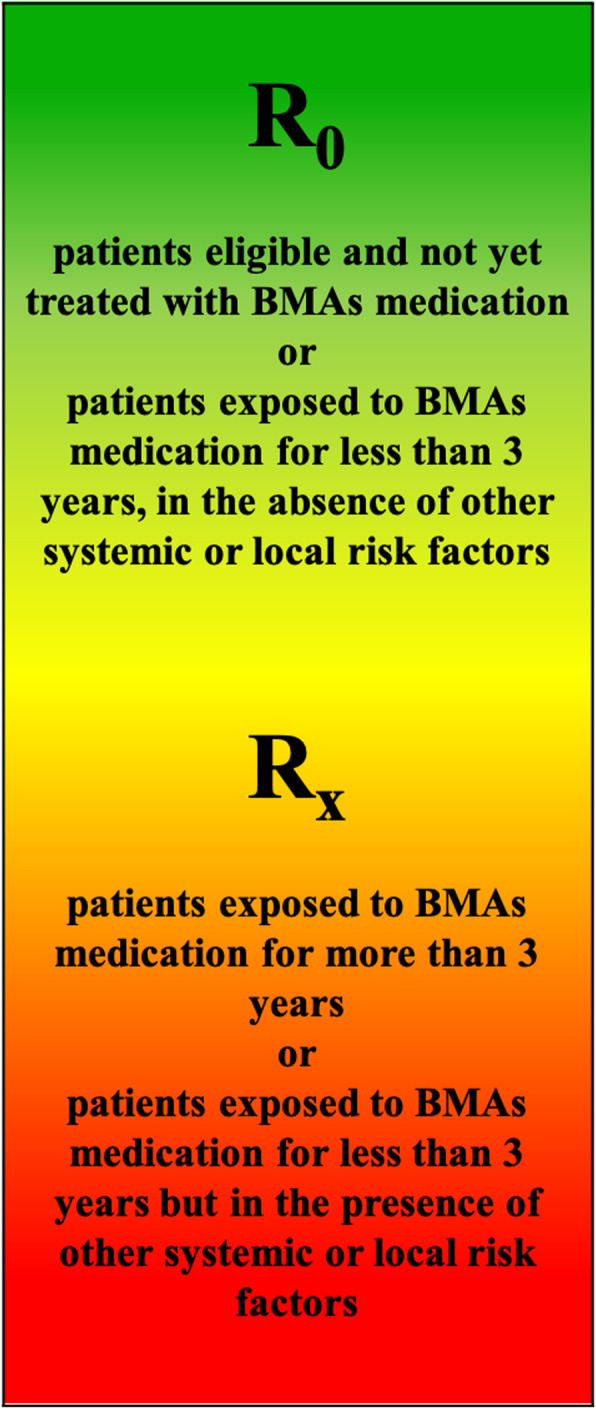

Medication-Related OsteoNecrosis of the Jaw (MRONJ) is associated with the use of bone modifying agents (BMAs) and, less commonly, anti-angiogenics [1, 2].

MRONJ is rarely observed in individuals with osteometabolic disorders (prevalence ranges between 0 and 0.4%), whereas it develops more commonly in metastatic cancer patients (0.2–6.7% prevalence) [3]. Although the etiopathogenesis of MRONJ remains unclear, the extreme inhibition of bone turnover together with local infection seem to represent the mechanisms of MRONJ development in patients receiving BMAs.

The length and cumulative dosage of antiresorptive therapy are associated with an increased risk of MRONJ development [4]. Furthermore, dental/periodontal infection and dentoalveolar surgery (e.g. dental extractions) have also been associated with MRONJ development [5].

As dental diseases are very prevalent and dental extractions are routinely and regularly performed in a large number of patients, risk-reduction strategies have been investigated with a view to minimise the risk of MRONJ development in individual who use BMAs and are in need of dental extractions.

The temporary discontinuation (i.e. drug holiday) of nitrogen-containing bisphosphonates (N-BPs) before dental surgery has become quite common in the clinical practice to minimise the risk of MRONJ. However, the effectiveness of a drug holiday not only has not been proved yet, but also lack a clear rationale based on the pharmacokinetics/pharmacodynamics of N-BPs. For these reasons, N-BPs discontinuation is not beneficial, at present [2, 3].

In the past years, the routine use of simple and surgical extraction of non-restorable teeth to eliminate dental/periodontal infections in patients with skeletal fragility undergoing N-BPs treatment was based on an individual risk-stratification [2, 6]. For example, alveoloplasty and primary wound closure have been proved successful and protecting in patients at increased risk of MRONJ [5].

However, some studies on alveoloplasty and primary wound closure were based on a one-fits-all approach, and did not attempt to identify and risk-stratify patients in groups at higher or lower risk of post-extraction MRONJ development [7].

Assessment of individual MRONJ risk profile (high risk vs low risk) becomes critical to select the appropriate dental treatment and defend patients from unnecessary (overtreatment) or deficient (undertreatment) interventions.

The cumulative risk of MRONJ in patients receiving BMAs for metabolic bone fragility increases with the time and reflects the rate of bone turnover suppression that largely depends on the dosage regimen and the duration of treatment; that risk is at least comparable for N-BPs and denosumab (DNB)(Fig. 1).

**Fig. 1:**
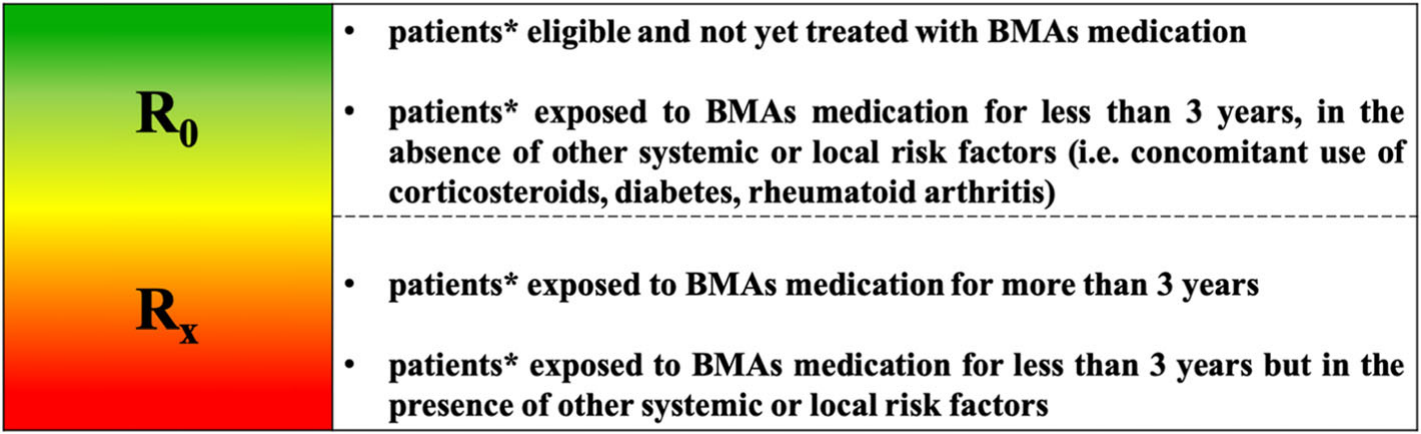
MRONJ risk profile of patients with metabolic bone fragility receiving Antiresorptive medications (BMAs)

While cumulative dosage plays a key role in the individual risk assessment of MRONJ in patients receiving N-BPs irrespective of the route of administration, this could not be true for patients receiving low-dose DNB (Prolia®). In fact, DNB does not bind to hydroxyapatite and incorporate into bone; thus, bone turnover recovers rapidly after drug suspension [8].

.As a result, interruption of DNB therapy before dental surgery is not advisable as it would expose osteoporosis patients to a rebound fracture risk [9].

.

Here we propose an alternative risk-reduction strategy for the osteoporosis patients receiving denosumab (Prolia®) that is based on the assessment of the patient’s individual risk associated with the pharmacokinetic/pharmacodynamic characteristics of the given medication.

The pharmacokinetics and pharmacodynamics of denosumab have been extensively investigated in multiple clinical studies [10–14]. Denosumab after a 60 mg subcutaneous dose was slowly absorbed, with peak serum concentration values generally reached within 4 weeks postdose. The serum concentration-time profile declined over a period of 4–5 months after the Cmax. The decline is biphasic, with an initial phase during which serum concentrations declined approximately linearly from peak followed by a more rapid terminal phase, with a serum concentrations mean half-life of approximately 25–30 days. At 24 months denosumab levels was similar to baseline level [10–13]. In addition, there has been no evidence of time-dependent pharmacokinetics after repeated dosing with the denosumab administered every 6 months (from 4 months to 4 years of exposure) [10, 12]. The reversibility of the pharmacodynamic effects (based on serum bone turnover markers) was observed at the end of the dosing interval [10, 14]. The mean serum CTX concentration decreased over 80% within 5 days after dosing; CTX starts to rise slowly from 4 months and at 6 months from the dose is up 10% compared to nadir [14].

The clinical relevance of body weight, race and age effects on pharmacokinetic parameters is probably limited, since a substantial overlap, as supported by pharmacokinetic and pharmacodynamic analysis where the covariate effect had a negligible impact [12, 13].

.

Accordingly, DNB pharmacokinetics suggest the presence of a window of opportunity between post-dose month 5 and 7, where recovery of bone turnover is initiated and dental extractions may be performed with a reduced risk of MRONJ development (Fig. 2).

**Fig. 2:**
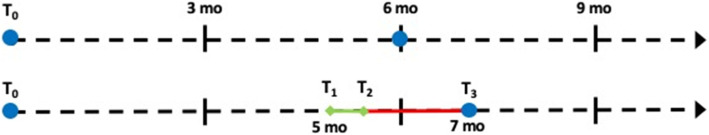
Dental management of patients at increased risk of fragility fractures receiving Prolia®

The recently acquired information that more than 7-month interval between 2 consecutive denosumab injections is likely to negatively impact on bone mineral density scores of osteoporosis patients during the off-treatment period, makes it practicable a 1-month delay of the subsequent dose of Prolia® to achieve mucosal healing following dental extraction [15, 16].

In order to adopt this strategy and benefit from this window of opportunity, dental practitioners should:

1. identify patients where dental surgery can be safely deferred to the 5th month following the last DNB dose;
2. communicate with the bone specialist (drug prescriber) and discuss the feasibility of delaying the following DNB dose by 1 month;
3. provide patients with exhaustive information about the possible risk and benefit of the planned procedure;
4. perform dental surgery based on routine dental care and strictly follow-up the healing process;
5. promptly communicate the progress of healing to the bone specialist, who will schedule the next dose of DNB.

In conclusion, we specul9ate that dental surgery may be safely performed without additional intra or peri-operative procedures in osteoporosis patients using DNB provided that careful case selection, adequate communication between health care providers is ensured and a 1-month deferral in the next DNB dose is feasible. Well-designed clinical trials are required to prove that this approach is effective while keeping on a low risk of MRONJ development.

## Data Availability

Data sharing is not applicable to this article as no datasets were generated or analyzed during the current study.

## Abbreviations

MRONJ: Medication-related osteonecrosis of the jaw
BMAs: Bone modifying agents
N-BPs: Nitrogen-containing bisphosphonates
DNB: Denosumab
